# Left ventricular functional assessment in murine models of ischemic and dilated cardiomyopathy using [^18^ F]FDG-PET: comparison with cardiac MRI and monitoring erythropoietin therapy

**DOI:** 10.1186/2191-219X-2-43

**Published:** 2012-08-03

**Authors:** Stefan Brunner, Andrei Todica, Guido Böning, Stefan G Nekolla, Moritz Wildgruber, Sebastian Lehner, Martina Sauter, Christopher Übleis, Karin Klingel, Paul Cumming, Wolfgang Michael Franz, Marcus Hacker

**Affiliations:** 1 Medical Department I, Ludwig-Maximilians-University, Klinikum Grosshadern, Marchioninistr 15, Munich, 81377, Germany; 2 Department of Nuclear Medicine, Ludwig-Maximilians-University, Klinikum Grosshadern, Marchioninistr 15, Munich, 81377, Germany; 3 Department of Nuclear Medicine, Technical University Munich, Klinikum Rechts der Isar, Ismaninger Str. 22, Munich, 81675, Germany; 4 Department of Radiology, Technical University Munich, Klinikum Rechts der Isar, Ismaninger Str. 22, Munich, 81675, Germany; 5 Department of Molecular Pathology, University of Tübingen, Liebermeierstr 8, Tübingen, 72076, Germany; 6 ABX GmBH, Heinrich-Glässer-Strasse 10-14, Radeberg, 01454, Germany

**Keywords:** Ejection fraction, Cardiomyopathy, Positron emission tomography, Magnetic resonance imaging, Erythropoietin

## Abstract

**Background:**

We performed an initial evaluation of non-invasive ECG-gated [^18^ F]FDG-positron emission tomography (FDG-PET) for serial measurements of left ventricular volumes and function in murine models of dilated (DCM) and ischemic cardiomyopathy (ICM), and then tested the effect of erythropoietin (EPO) treatment on DCM mice in a preliminary FDG-PET therapy monitoring study.

**Methods:**

Mice developed DCM 8 weeks after injection with Coxsackievirus B3 (CVB3), whereas ICM was induced by ligation of the left anterior descending artery. LV volumes (EDV and ESV) and the ejection fraction (LVEF) of DCM, ICM and healthy control mice were measured by FDG-PET and compared with reference standard results obtained with 1.5 T magnetic resonance imaging (MRI). In the subsequent monitoring study, LVEF of DCM mice was evaluated by FDG-PET at baseline, and after 4 weeks of treatment, with EPO or saline.

**Results:**

LV volumes and the LVEF as measured by FDG-PET correlated significantly with the MRI results. These correlations were higher in healthy and DCM mice than in ICM mice, in which LVEF measurements were somewhat compromised by absence of FDG uptake in the area of infarction. LV volumes (EDV and ESV) were systematically underestimated by FDG-PET, with net bias such that LVEF measurements in both models of heart disease exceeded by 15% to 20% results obtained by MRI. In our subsequent monitoring study of DCM mice, we found a significant decrease of LVEF in the EPO group, but not in the saline-treated mice. Moreover, LVEF in the EPO and saline mice significantly correlated with histological scores of fibrosis.

**Conclusions:**

LVEF estimated by ECG-gated FDG-PET significantly correlated with the reference standard MRI, most notably in healthy mice and mice with DCM. FDG-PET served for longitudinal monitoring of effects of EPO treatment in DCM mice.

## Background

Murine models of cardiovascular disease are increasingly important for evaluation of novel therapeutic approaches. A primary endpoint in such models is left ventricular ejection fraction (LVEF), which is a strong independent predictor of cardiovascular morbidity and death [1,2]. Several techniques have been developed for the assessment of left ventricle (LV) function in small animal investigations. Pressure-volume relations can be measured with a conductance microcatheter, with the caveat that this invasive method is not permissive to follow-up examinations in small animals [3]. Echocardiography is widely used in mice, despite its high intra- and inter-observer variability [4]. In the clinical setting, cardiac magnetic resonance imaging (MRI) and blood pool single-photon emission computed tomography are considered as gold standards for the assessment of LVEF [5-7], but have not found wide use for monitoring cardiac therapies in mice.

As an alternative to these approaches, positron emission tomography (PET) with the glucose analogue ^18^ F]-fluorodeoxyglucose (FDG), allows analysis of cardiac function and myocardial metabolism within the same setting [8,9]. Indeed, FDG-PET has been evaluated against reference standard MRI methods for the calculation of LV function in healthy mice and in mice with ischemic cardiomyopathy (ICM) due to myocardial infarction (MI) [10-12], but has not yet been tested in murine models of dilative cardiomyopathy (DCM). Furthermore, the fitness of the applicability of gated FDG-PET for therapeutic monitoring in mouse models of myocardial diseases has not been established. Therefore, we compared the accuracy of ECG-gated FDG-PET LVEF measurements in healthy mice, and in murine models of ICM and also in DCM-induced by Coxsackievirus B3 (CVB3) exposure. Quantitative results were compared with reference standard LV-function measurements obtained with 1.5 T clinical MRI. We then proceeded to test gated FDG-PET for longitudinal monitoring in mice with CVB3-induced DCM after erythropoietin (EPO) treatment, based upon previous reports of its cardioprotective properties in preclinical models of myocardial infarction [13,14] as well as in rats with autoimmune-induced myocarditis [15,16].

## Methods

### Animal models

To obtain a murine model of ICM, MI was induced in (*N* = 7) male C57BL/6 wildtype (WT) mice (Charles River Laboratories, Sulzbach, Germany) by surgical occlusion of the left anterior descending artery (LAD), as described previously [17]. Mice were examined by PET and MRI 4 weeks after MI. To obtain a murine model of DCM, SWR/J (H-2q) mice (*N* = 11) were infected with CVB3 via intraperitoneal injection (1 × 10^5^ pfu [5]) [18]. This procedure provokes mice to develop acute myocarditis at 6 to 12 days after injection, and subsequently to proceed to a chronic phase of myocarditis, resulting several weeks later in a DCM phenotype [19]. Our DCM mice were examined by PET and MRI 12 weeks after infection, with healthy, age-matched SWR/J (H-2q)-mice (*N* = 4) serving as controls. For all imaging examinations, anaesthesia was induced with isoflurane (2.5%) and maintained throughout the examination with isoflurane (1.5%) delivered in oxygen (1.2 L/min) via a mask. Animal care and all experimental procedures were performed in strict accordance to the Guide for the Care and Use of Laboratory Animals published by the US National Institutes of Health (NIH publication no. 85–23, revised 1996) and was approved by the local animal care and use committees.

### Administration of EPO

In a separate intervention/treatment study, SWR/J (H-2q) mice (*N* = 11) with CVB3-induced DCM underwent a baseline FDG-PET examination 8 weeks after CVB3 infection. Following this scan, mice were randomised into groups which were treated thrice weekly for 2 weeks with subcutaneous injections of saline (*N* = 5) or EPO (2000 IU/kg; erythropoietin alpha, Janssen-Cilag, Neuss, Germany; *N* = 6). Follow-up scans were performed 2 weeks after completion of the treatment, thus 4 weeks after the baseline scans.

### Cardiac magnetic resonance imaging

For reliable ECG synchronisation and high resolution imaging, a dedicated small animal ECG device, 1025-MR (SA Instruments Inc., Stony Brook, NY), with a microscopy coil (Philips Medical Systems, Best, NL) was used. Imaging was performed on a 1.5 T Philips Achieva MR scanner using a clinical gradient system (30 mT/m, 150 mT/m/ms). The mice were imaged in prone position with the thorax placed on top of the microscopy single loop surface coil (*D* = 2.3 mm). High-resolution MRI sequences for assessment of myocardial function and morphology were implemented as described previously [9]. In brief, cine MRI was performed with prospective ECG gating using a spoiled gradient echo technique. Imaging parameters included TR/TE = 18 ms/6.5 ms, flip angle = 30°, averages = 1, FOV = 35 mm, matrix = 128, resulting in a spatial resolution of 0.22 × 0.22 × 1 mm at a temporal resolution of 18 ms.

Quantitative analysis of the cine MRI was performed using a semi-automated approach employing a dedicated software package (Munich Heart/MRI®, Technical University Munich, Germany) [9]. The end, epi- and endocardial contours of the entire LV slices were manually traced at end-diastolic (EDV) and end-systolic (ESV) phases for calculation of the corresponding left ventricular volumes, as well as the LVEF.

### Cardiac PET imaging

PET was performed using the Siemens Inveon P120 PET scanner (Siemens Healthcare Molecular Imaging, Knoxville, TN, USA). After placing a catheter into a tail vein, the mice were positioned in a prone position within the aperture of the PET scanner and were kept warm with a heating pad so as to maintain core body temperature within the normal range, with continuous measurement of rectal temperature. Cardiac excitation and respiration were measured and recorded throughout the scan using a dedicated system (BioVet; Spin Systems Pty Ltd., USA). ECG electrodes were placed on both forepaws and the left hindpaw, and respiration was measured with a small pressure detector lying under the mouse thorax. After intravenous administration of FDG (19 ± 5 MBq), an ECG-gated emission recording followed throughout the interval 60 to 90 min after tracer injection and concluded with a 7-min transmission scan for attenuation correction. Animals were returned to their home cage for recovery. The cardiac cycle from the FDG list-mode acquisitions was divided into eight equal intervals using the Siemens-Inveon acquisition workplace and reconstructed using MAP 3D with 32 iterations and OSEM 3D with 4 iterations. Since we used eight bins for gated reconstruction, the time resolution for the PET is equal to the mean heartbeat duration/8 for a mean heart rate of 500 bpm resulted in a temporal resolution of 15 ms. Each image volume consisted of 128 × 128 × 159 voxels of size 0.4 mm × 0.4 mm × 0.8 mm, resulting in a volumetric resolution of approximately 1.5 μL [20]. Final pixel size was magnified by a factor of ten as required for compatibility of mouse data with QGS software tools [21]. EDV, ESV and LVEF measurements from the PET data sets were calculated using automated software packages (QGS 2008 [22,23], Cedars Sinai Medical Center, Los Angeles, USA). The automated wall recognition proved to be robust, such that no further manual interventions were required.

### Infarct size

Infarct sizes were estimated from FDG-PET data as percentage of the left ventricular myocardium area. To this end, the entire 30 min recording was reconstructed (MAP 3D with 32 iterations and OSEM 3D with 4 iterations in a 128 × 128 matrix) as a static image and analysed using MunichHeart® (Technical University Munich, Germany) with manual definition of the long axis of the resultant polar map. The infarct area was delineated with an intensity threshold 60% of the activity in a septal ROI [24,25].

### Histology

To evaluate the diagnosis of CVB3-induced DCM histopathologically, the hearts were removed 12 weeks after infection and fixed in phosphate buffered 4% formalin prior to blocking of 2-mm thick slices, which were embedded in paraffin. Five micrometre-thick sections were cut and stained with Masson trichrome. We quantified the extent of fibrotic lesions in picrosirius red stained sections according to a score ranging from 0 to 4, as previously described [26].

### Statistical analyses

Data was analysed using Predictive Analysis Software 19. Mean values and standard deviations (SD) for EDV, ESV and LVEF were calculated. Means obtained by PET and MRI were compared using paired Student' *t* test. The agreement between the methods was visualised using Bland-Altman plots [27]. Additionally, Pearson correlation coefficients (*R*) and linear regressions were calculated and performed. Values of *P* < 0.05 were considered statistically significant.

## Results

### Estimation of left ventricular functional parameters

**Figure 1:**
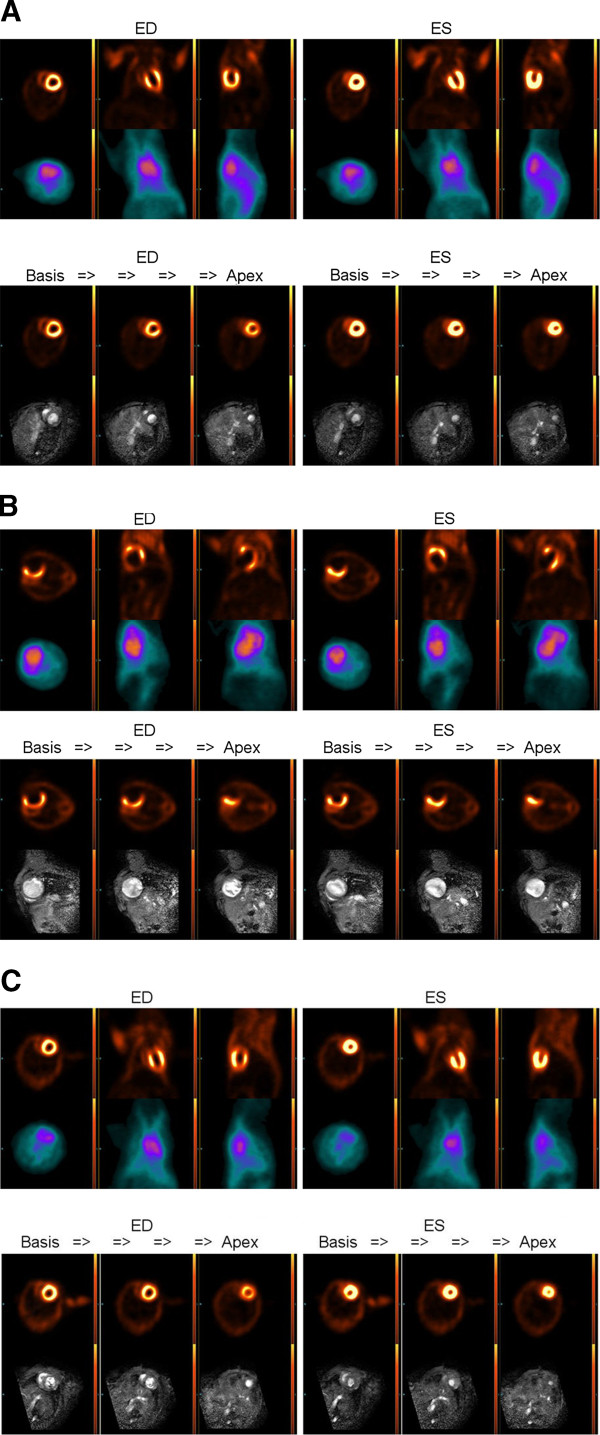
**Comparison between different imaging modalities, representative images.** FDG-PET versus MRI (lower row) for animals with DCM (**A**, LVEF: MRI = 26%, FDG-PET = 37%), ICM (**B**, LV-EF: MRI = 12%, FDG-PET = 34%) and for healthy control animals (**C**, LVEF: MRI = 48%, FDG-PET = 68%).

**Figure 2:**
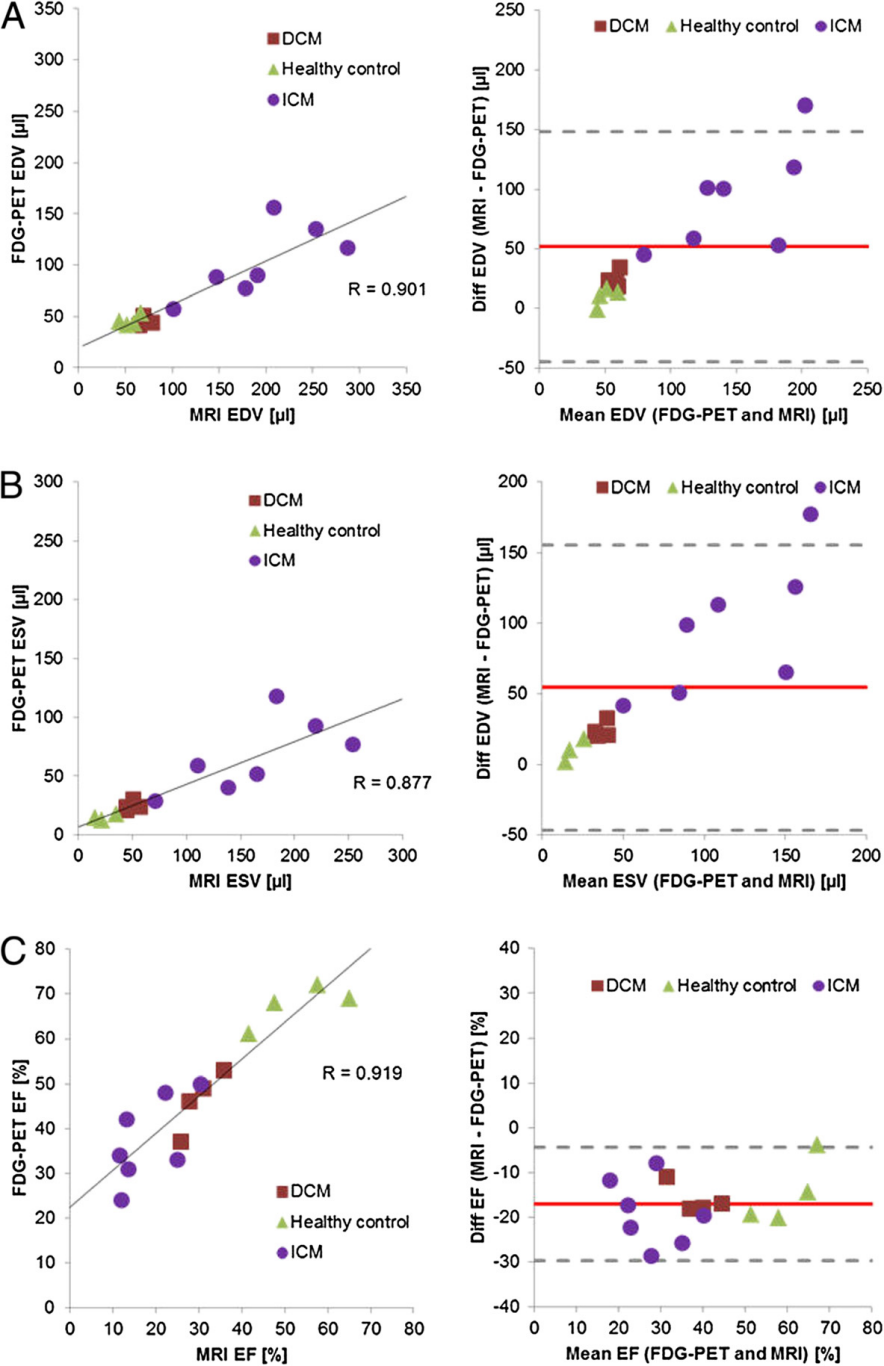
**Scatter plots of the relationship between measurements of LV parameters.** Correlations between LV-functional parameter measurements within the entire study group by FDG-PET and by MRI for all animals (left hand side). alongside the corresponding Bland-Altman-plot (right hand side) for all animals, for the cases of (**A**) EDV, (**B**) ESV and (**C**) LVEF.

Figure 1 shows representative end-diastolic and end-systolic images of FDG-PET and MRI from the myocardial base to the apex for animals with DCM (A), ICM (B) and for healthy control (C) animals. There were high correlations between LV-functional parameter (EDV, ESV and LVEF) measurements within the entire study group (Figure 2A,B,C). Results summarised in Table 1 show that FDG-PET significantly underestimated the magnitudes of EDV and ESV and significantly overestimated the LVEF, as compared to our reference standard cine MRI. Bland-Altman plots confirmed the underestimation of EDV and ESV, as well as the overestimation of LVEF by FDG-PET relative to MRI, but nearly all difference values fell within ± 1.96 SD (Figure 2A,B,C right hand side, Table 1). Considering only control mice together with the DCM group, the bias was even lower and the limits of agreement in the Bland-Altman plots were even more stringent (Table 1).

**Table 1:** Comparison between LV-functional parameters as calculated by cine MRI and FDG-PET for different study subgroups

	**HC + DCM + ICM**		**HC + DCM**		**ICM**	
		**Cine MRI**	**FDG-PET**	**Cine MRI**	**FDG-PET**	**Cine MRI**	**FDG-PET**
EDV (μL)	125 ± 80	72 ± 37*	63 ± 11	46 ± 4*	195 ± 63	103 ± 35*
Bias		52		17		92
LoA		−44 to 149		−3 to 37		5 to 180
ESV (μL)	96 ± 77	42 ± 32*	38 ± 14	20 ± 6*	163 ± 63	67 ± 31*
Bias		54		18		96
LoA		−47 to 155		0 to 36		3 to 190
LVEF (%)	31 ± 16	48 ± 15*	42 ± 14	57 ± 13*	18 ± 7	37 ± 10*
Bias		−17		−15		−19
LoA		−30 to −4		−26 to −5		−34 to −5

Values are presented as mean ± standard deviation for the comparison of FDG-PET with the reference method cine MRI. Furthermore the corresponding biases and limits of agreement (LoA) calculated from Bland-Altman plots are presented. *Student's *t* test revealed a statistically significant difference.

### Correlation between infarct size and ejection fraction

**Figure 3:**
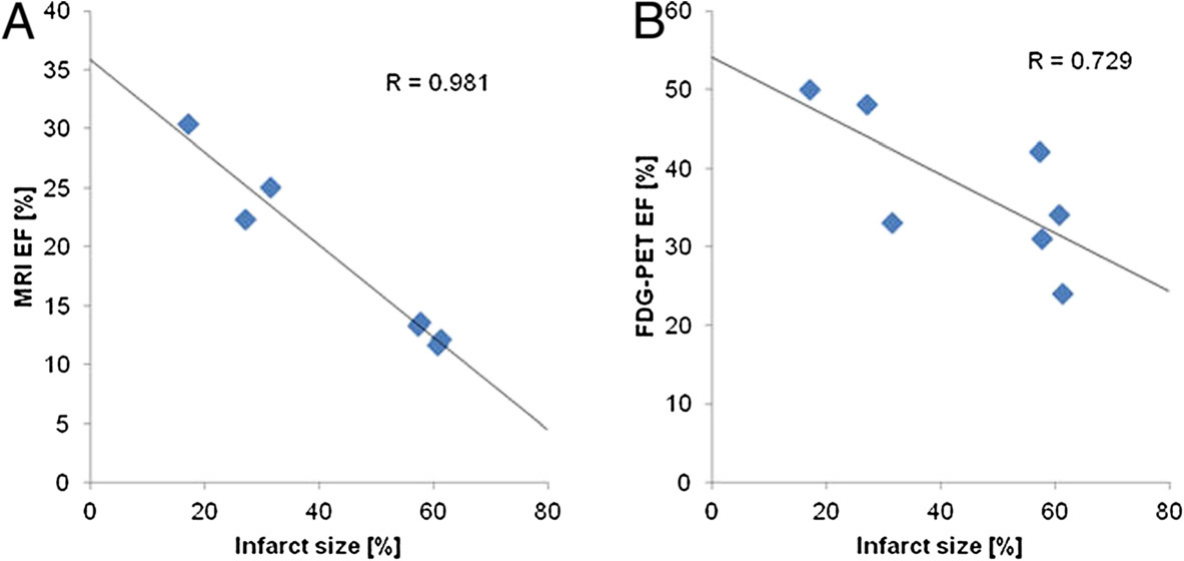
Correlation between infarct size and LVEF measured by MRI (A) and FDG-PET (B).

There was a high negative correlation found between the infarct sizes in the FDG images from the ICM group and the LVEF values as measured by MRI (*R* = 0.98), and a lesser correlation between the infarct sizes and the LVEF derived from FDG-PET (*R* = 0.73) (Figure 3).

### Monitoring LVEF in DCM mice treated with erythropoietin

**Figure 4:**
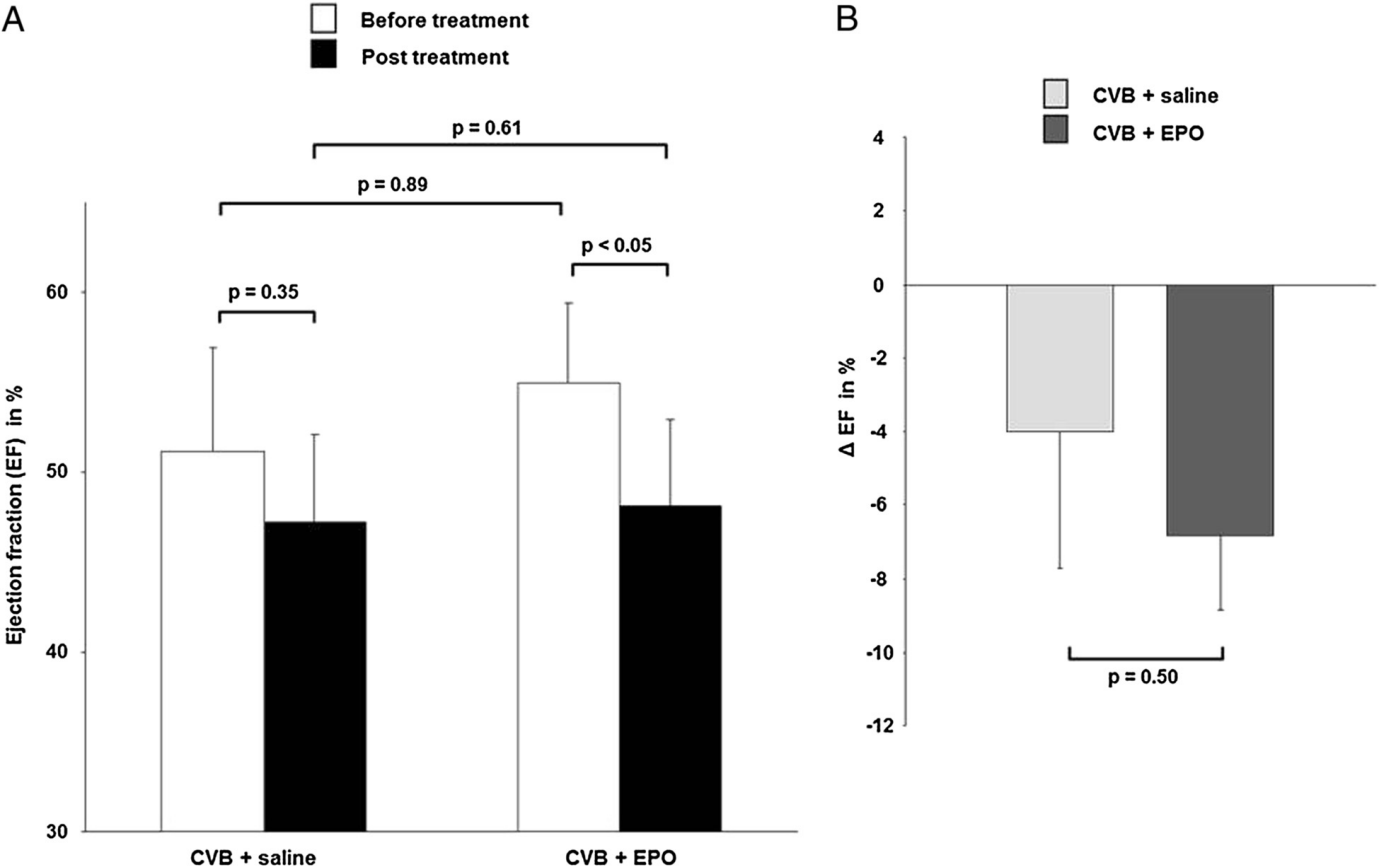
**Monitoring EPO treatment with ECG-gated FDG-PET.** (**A**) The mean magnitude of LVEF before (white bars) and after (black bars) treatment with saline vs. EPP, and (**B**) the change in LVEF (Δ EF) for the saline and EPO groups during 1 month of treatment.

**Figure 5:**
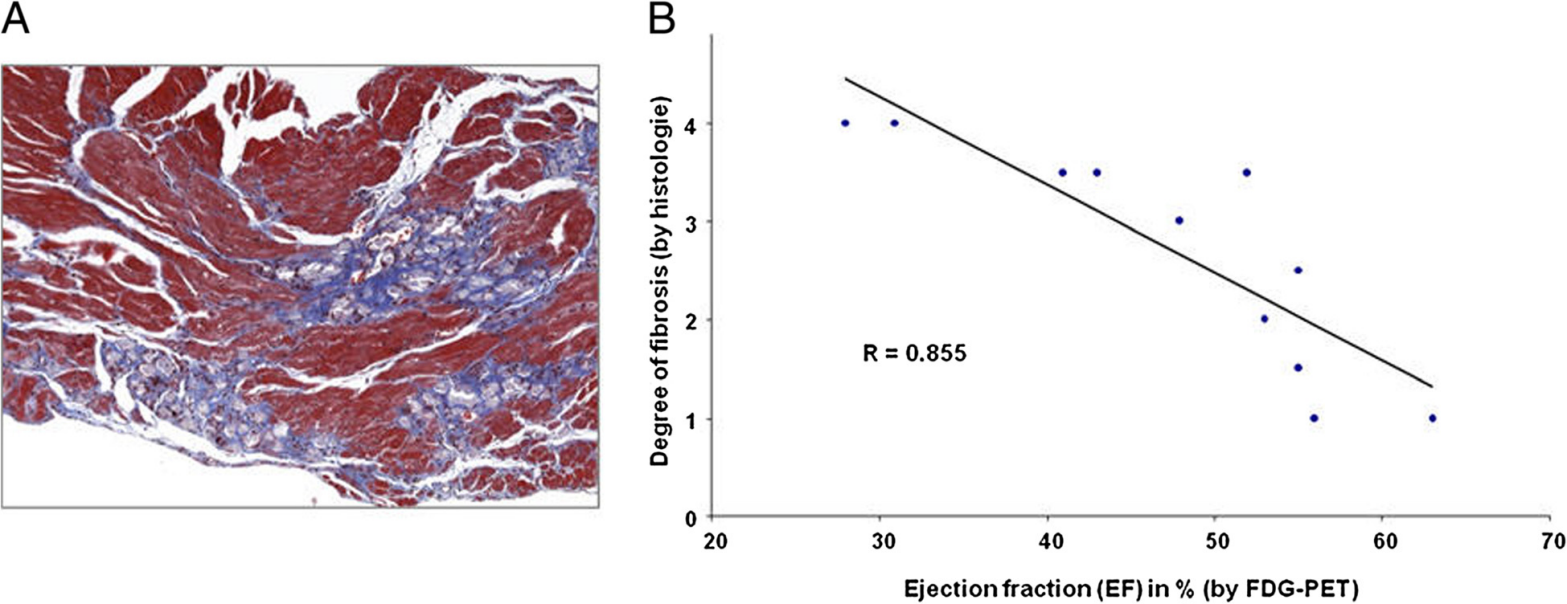
**Correlation of LVEF as assessed by ECG-gated FDG-PET with degree of fibrosis.** (**A**) Representative histological cardiac findings showing myocardial fibrotic areas in CVB3-infected mice (Masson trichrome staining). (**B**) Correlation of degree of fibrosis obtained histology with LVEF measured by FDG-PET.

Means and standard deviations for EDV, ESV, stroke volume (SV) and LVEF for the baseline and follow-up measurements, along with the corresponding *p* values for treatment effects are summarised in Table 2. In the EPO-treated group, there was a significant decrease in the magnitude of LVEF from 55 ± 11% to 48 ± 12% (*p* = 0.02), whereas the ESV significantly increased from 22 ± 8 μL to 26 ± 10 μL (*p* = 0.012). The EDV showed a significant increase from 40 ± 9 μL to 52 ± 15 μL in the saline-treated group (*p* = 0.031). For all other functional parameters, no significant changes were observed in the saline- and EPO-treated groups. There were no significant differences in LVEF observed between EPO- and saline-treated mice before and after treatment (Figure 4A). Although the Student's *t* test revealed a significant decrease for LVEF in the EPO-treated group, the difference of the LVEF degradation between the saline and the EPO groups fell just short of being significant (*p* = 0.050; Figure 4B). The fibrosis scores in these mice correlated with the LVEF measured by FDG-PET (*R* = 0.86) (Figure 5).

**Table 2:** LV–function parameters measured at baseline and after 4 weeks with FDG-PET

**EPO (*N*** **= 6)**	**Baseline**	**Follow-up**	***P***	**Saline (*N*** **= 5)**	**Baseline**	**Follow-up**	***P***
EDV	47 ± 7	49 ± 9	0.371	EDV	40 ± 9	52 ± 15	0.031
ESV	22 ± 8	26 ± 10	0.012	ESV	20 ± 10	29 ± 16	0.078
SV	25 ± 2	23 ± 5	0.462	SV	20 ± 3	23 ± 1	0.064
EF	55 ± 11	48 ± 12	0.020	EF	51 ± 13	47 ± 11	0.345

Values are presented as mean and standard deviation, along with the corresponding *p* values (Student's *t* test).

## Discussion

Quantification of left ventricular functional parameters in murine models of heart disease entails considerable technical difficulties, arising from the small size of the heart and its rapid movement, with heart rates typically around 500 bpm. In the present study, we conducted a comparison of different LV-function quantification methods in groups of healthy mice and in two models of cardiomyopathy. We compared FDG-PET measurements acquired on a dedicated small animal PET system with the reference standard for LV-function measurements, i.e. cine MRI, which was performed with a 1.5 T clinical scanner equipped with a microscopy coil. In the entire study group, we found high correlations between FDG-PET and the reference standard LV-function measurements, albeit with a systematic underestimation of the LV volumes, propagating to a net overestimation of the LVEF by FDG-PET. Due to imperfect delineation of myocardial scar tissue, the bias between FDG-PET and the reference standard results was markedly higher in the ICM mice.

To our knowledge, this is the first investigation examining the fitness of ECG-gated FDG-PET for the estimation of LV function in different mouse models of cardiovascular disease within one setting. In an earlier proof-of-principal study conducted in only two healthy mice, Yang et al. demonstrated the technical possibility of measuring murine LVEF with cardiac-gated FDG-PET [11]. In another study, gated FDG-PET was validated against echocardiography in rats with myocardial infarction [21]. Stegger et al. were the first to quantify LV volumes and ejection fraction using FDG-PET compared to MRI [12], both in healthy mice and in mice with permanent (ICM) or transient LAD ligation. In contrast to the data from human studies and to the results of [12], we observed a significant overestimation of the LV volumes. Furthermore, compared to their LVEF values in healthy mice (68 ± 6% in PET and 66 ± 4% in cine MRI), mean LVEF estimates in our mixed group of healthy control and DCM mice (57 ± 13% in PET and 42 ± 14% in cine MRI), which were pooled so as to obtain adequate statistical power for this comparison, stand somewhat in contrast to higher corresponding results reported by [12]. However, our decision to combine the healthy and DCM groups does not account for the present finding of a 25% overestimation of LVEF by FDG-PET as compared to MRI. Our finding of lower LVEF by MRI can likely be attributed to our use of a clinical 1.5 T MRI, whereas [12] used a 6.3 T dedicated small animal MRI, which provides images less vulnerable to partial volume effects, and in more slices. Furthermore, we used a semi-automated approach for the quantitative analysis of the cine MRI (MunichHeart/MRI®, Technical University Munich, Germany) [9] wherein epi- and endocardial contours of the entire left ventricle slices were manually traced at end-diastolic and end-systolic phases. Consequently, the definition of the heart base is somewhat observer-dependent. Since we acquired seven MR slices for the whole mouse heart, imperfect definition of the valve plane could have had an impact on the final calculation of the corresponding left ventricular volumes as well as on the LVEF. This limitation could partially account for the bias between our FDG-PET measurements relative to cine MR. For the present, dedicated animal MRI systems are only available at a few imaging laboratories, whereas clinical MRI tomographs are widely available and provide a mature and validated imaging platform for animal imaging, albeit with certain limitations noted above.

Similarly, our FDG-PET measurements of LVEF in ICM mice (37 ± 10%) match the results presented by Stegger et al. [12] (32 ± 8%) but with an even greater discrepancy (nearly two-fold) between the FDG-PET and MRI measurements. Here, another factor contributing to our discrepant findings compared to the results of [12] is the time-point of examination. Whereas [12] examined their mice 2 weeks after myocardial infarction, our animals were scanned after 4 weeks, a delay which probably led to more advanced dilation of the left ventricle, which could have hindered delineation of the endocardial borders in the FDG images, propagating to a systematic overestimation of LVEF in our study. This conjecture is supported by the fact that mean volumes measured by MRI were much higher in our ICM group (EDV, 195 ± 63; ESV, 163 ± 63) compared to the findings by [12] (EDV, 122 ± 65; ESV, 94 ± 67).

There is a general consensus arising from human studies of LV function that values obtained with different segmentation algorithms are not perfectly interchangeable but require scaling between methods [28]. The Cedars tools (QGS®; Cedars-Sinai Medical Center, Los Angeles, CA) have already been used to evaluate LV function in rodents and showed physiologically reasonable results, as noted above. Nonetheless, the different valve plane definitions could explain both our underestimation of the LV volumes and the net effect of overestimation of the LVEF by FDG-PET. Notwithstanding the advantages imparted by cine MRI measurements, FDG-PET, in addition to LV functional parameters, provides reliable quantification of infarct sizes within the same setting. Indeed, the parameter LVEF and LAD infarct sizes are inextricably related, as demonstrated by the very high negative correlations seen in the present study (Figure 3), which exemplifies the extent to which heart output declines with increasing area of infarcted LV.

The somewhat lower correlation between LVEF measurements by FDG-PET and reference standard methods in the ICM group should not mitigate against the use of FDG-PET in the present DCM model. Therefore, we chose to use FDG-PET in the EPO-treatment therapy monitoring arm of this study. The sensitivity of functional FDG-PET in this context is made clear by the high correlation between the reduction in LVEF and the degree of histological fibrosis (Figure 5B), which is an indicator of the extent of pathological remodelling of the myocardium. EPO is a growth factor exerting effects at diverse targets in addition to its well-known classical action to stimulate erythrocyte production. We have earlier found EPO to exert cardio-protective effects due to intrinsic anti-apoptotic properties and also due to mobilisation of bone marrow-derived stem cells [13,14,29,30]. The first clinical trial of EPO in patients with chronic heart failure gave promising findings of increased exercise capacity and cardiac function [31,32]. However, clinical trials of EPO treatment after myocardial infarction revealed discrepant results [33-35] presumably due to a trade-off between actual benefits reflecting improved cardiac function versus correction of incidental anaemia, further confounded by deleterious effects arising from increased haematocrit. In previous reports in a rodent model of myocarditis [15,16], EPO treatment produced substantial reductions in macrophage infiltration and necrosis while rescuing cardiac function when administered soon after the onset of autoimmune pathology. However, in the present study of mice with established CVB3-induced DCM, the EPO treatment seemingly resulted in a significant decrease of LVEF. Our saline-treated DCM mice showed a strong trend towards decreasing LV function to follow-up, but without statistical significance, such that there emerged no notable difference between the two treatment groups. The apparently deleterious effect of EPO treatment on LVEF in DCM mice may be due to the high dose used in the present study, which may have induced polycythaemia, resulted in impaired cardiac function. As such, negative effects of EPO seem to have predominated over protective effects reported in other studies.

## Conclusions

In conclusion, LVEF in murine hearts measured by ECG-triggered FDG-PET correlates highly with gated MRI measurements. However, the FDG method systematically overestimated LVEF relative to the gated MRI gold standard. Despite this limitation, FDG-PET served for monitoring effects of EPO treatment on cardiac function in mice with virus-induced dilatative cardiomyopathy.

## Competing interests

The authors declare that they have no competing interests.

## Authors' contributions

SB and AT made the conception and design, as well as the analysis and interpretation of data. GB conducted the analysis of data. SN has done the analysis of data and revision of the manuscript critically for important intellectual content. MW, SL and CÜ did the analysis and interpretation of data. MS carried out the DCM animal models and interpretation of data. KK is responsible for the animal models and interpretation of data. PC did the revision of the manuscript critically for important intellectual content. WF did the revision of the manuscript critically for important intellectual content and the final approval of the manuscript. MH made the conception and design, as well as analysis and interpretation of data, revising the manuscript critically for important intellectual content and final approval of the manuscript. All authors read and approved the final manuscript.
